# Bringing on the itch

**DOI:** 10.7554/eLife.52931

**Published:** 2019-11-29

**Authors:** Daniel A Waizman, Sourav Ghosh, Carla V Rothlin

**Affiliations:** 1 Department of Immunobiology, Yale School of MedicineYale UniversityNew HavenUnited States; 2 Department of Neurology, Yale School of MedicineYale UniversityNew HavenUnited States

**Keywords:** chronic itch, neutrophils, somatosensory, neuroimmune, itch, atopic dermatitis, Mouse

## Abstract

Neutrophils are the first immune cells that enter the skin and cause itch in atopic dermatitis.

**Related research article** Walsh CM, Hill RZ, Schwendinger-Schreck J, Deguine J, Brock EC, Kucirek N, Rifi Z, Wei J, Gronert K, Brem RB, Barton GM, Bautista DM. 2019. Neutrophils promote CXCR3-dependent itch in the development of atopic dermatitis. *eLife* **8**:e48448. doi: 10.7554/eLife.48448

Atopic dermatitis is characterized by debilitating chronic itch and affects about 12% of children and 7% of adults in the United States (Silverberg, 2017). Most itching is harmless, and it can act as a defense mechanism when it triggers scratching behaviors that remove macroscopic parasites or harmful substances from the skin. But the itch associated with atopic dermatitis is far from harmless, being linked to loss of sleep, reduced quality of life and psychiatric symptoms (Chiesa Fuxench et al., 2019).

To date, the most successful treatment for atopic dermatitis is dupilumab, an antibody that regulates two signaling pathways (the IL-4 and IL-13 pathways) by binding to a receptor called IL-4Ra. In clinical trials of adult patients with moderate-to-severe atopic dermatitis, approximately half showed a reduction in the severity of the itch after a year of dupilumab injections and treatment with corticosteroids (Blauvelt et al., 2017). While this confirms that IL-4Ra has a role in causing the itch associated with atopic dermatitis, the fact that a significant number of patients did not respond to treatment makes it clear that our understanding of this condition is far from complete. Now, in eLife, Diana Bautista (University of California, Berkeley) and colleagues – including Carolyn Walsh and Rose Hill as joint first authors – report that white blood cells called neutrophils also have a central role in atopic dermatitis (Walsh et al., 2019).

Walsh et al. exposed mice to calcipotriol, a chemical that induces symptoms similar to human atopic dermatitis, and analyzed its effect on the behavior of the mice, and also its effect at the cellular and molecular level. Calcipotriol is known to induce the production of TSLP, a cytokine that activates several types of immune cells (including CD4^+^ T cells, eosinophils and mast cells). These cells are commonly associated with itch, which leads to scratching behavior in mice (Li et al., 2006; Yoo et al., 2005; Mack and Kim, 2018).

The experiments of Walsh et al. showed that neutrophils infiltrated the exposure site before any of the other immune cells associated with the response to TSLP. Neutrophil infiltration occurred at the same time as the mice started scratching the site of calcipotriol application, a behavior that was significantly reduced when neutrophils were depleted. Furthermore, injecting a chemokine that recruits neutrophils into mice that had not been exposed to calcipotriol was sufficient to induce scratching behavior. These results indicate that neutrophils may be causing some of the itch associated with atopic dermatitis.

Next, Walsh et al. used mice that had been genetically modified to lack the TSLP receptor (TSLPR), also called TSLPR knock-out mice. When these mice were exposed to calcipotriol, fewer basophils, CD4^+^ T cells and mast cells were recruited to the exposure site, confirming that TSLP had a role in recruiting these itch-inducing cells. However, the knock-out mice still exhibited scratching behavior in the early stages of calcipotriol application, even though the behavior decreased significantly later on. This suggests that TSLP has a role in causing itch in later stages of atopic dermatitis, but other mechanisms must be responsible for causing itch early on. Neutrophil infiltration was not affected in TSLPR knock-out mice. These results indicate that neutrophils have an early (as well as a sustained) role in the progression of the itch associated with atopic dermatitis.

Analyzing the gene expression of mice exposed to calcipotriol showed that genes affecting how the skin acts as a barrier changed quickly after exposure, followed by alterations in neuronal and cytokine genes. The activity of some cytokine genes increased when both neutrophils and TSLPR were present, while others were upregulated independently of neutrophils but dependent on TSLPR. Yet other genes coding for cytokines such as CXCL10 depended on the presence of neutrophils but not TSLPR.

CXCL10 is a cytokine that can be produced by neutrophils and is known to signal through CXCR3 in sensory neurons to drive scratching behavior (Qu et al., 2015). Walsh et al. showed that blocking CXCR3 attenuated both the early and late scratching behavior induced by calcipotriol. Thus, early infiltration by neutrophils may induce innervation of the affected skin and sensitize the neuronal circuits responsible for scratching behavior by signaling through CXCL10 and CXCR3.

The work of Walsh et al. reveals a new mechanism contributing to the itch associated with atopic dermatitis and reinforces the concept that there are multiple drivers in this disease. Along with TSLP and IL4/IL-13, CXCL10 may cause some of the symptoms of atopic dermatitis, although the role of this cytokine in the disease remains to be fully characterized. Importantly, neutrophils and TSLP represent independent pathways in the chronic phase of calcipotriol treatment in mice (Figure 1). This suggests that a subset of human patients with atopic dermatitis may be more sensitive to TSLP, and another more sensitive to neutrophils. This neutrophil-sensitive group of patients may benefit from treatments that interfere with neutrophil activity or with the communication between neutrophils and neurons through CXCL10/CXCR3.

**Figure 1.**
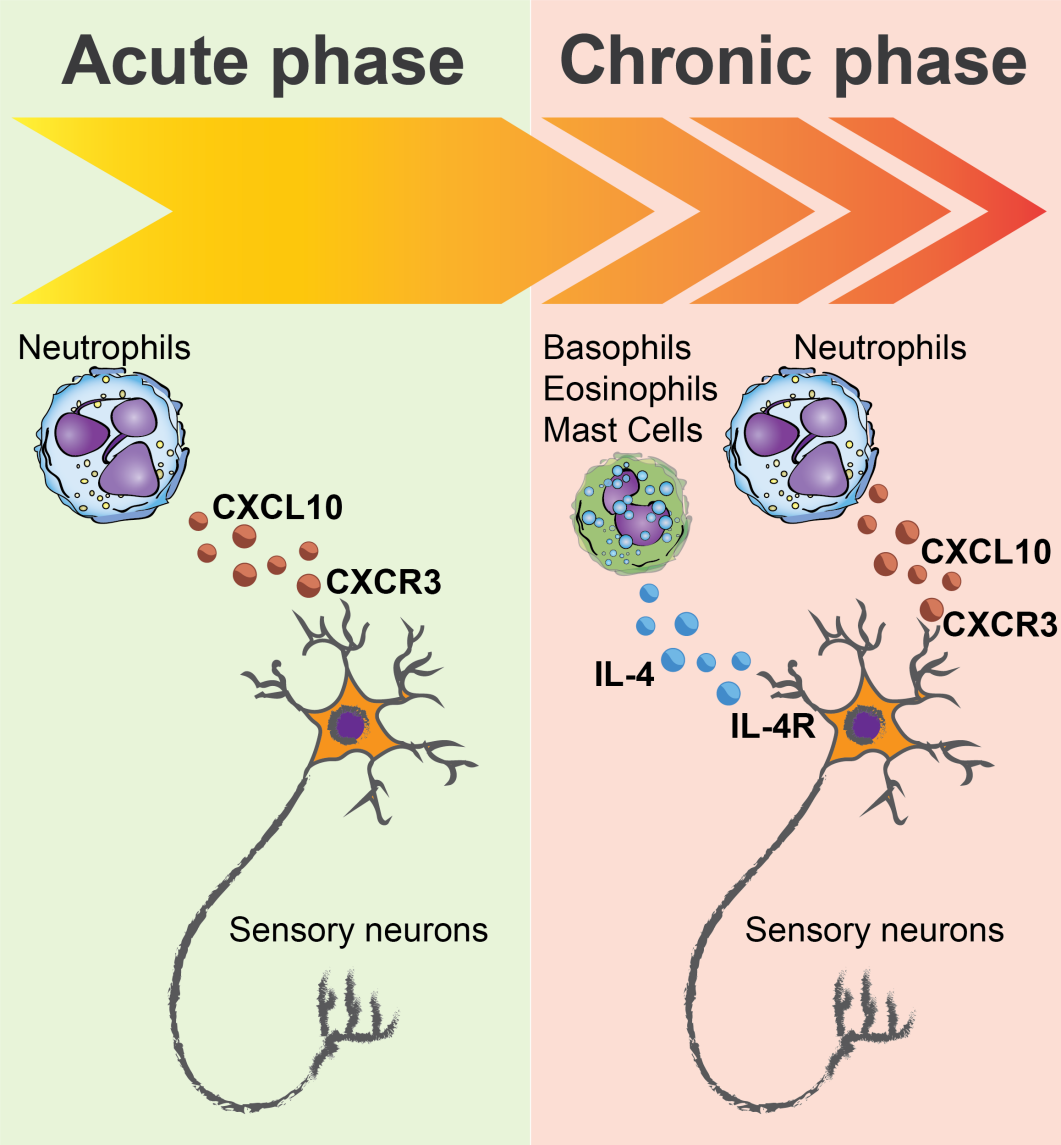
Neutrophils are essential players in itch. Exposing mice to a chemical called calcipotriol leads to itching and scratching behavior similar to that observed in humans with atopic dermatitis. Walsh et al. observed prominent infiltration by immune cells called neutrophils at the site where the calcipotriol had been administered during both acute (early) itch and chronic (late) itch. In the acute phase (left) neutrophils led to the production of the cytokine CXCL10, which signals through the receptor CXCR3 to drive itch. CXCR3 is a receptor expressed on the surface of sensory neurons in the skin called nociceptors. In the chronic phase (right), a cytokine called TSLP (not shown) acts with neutrophils to cause itch. The presence of TSLP leads to infiltration by other immune cells (including basophils, eosinophils and mast cells) that are known to contribute to itch. The presence of TSLP also leads to the production of IL-4, which binds to the IL-4 receptors (IL-4R) on nociceptive neurons, making these neurons more likely to respond to histamine and other signals that cause itch.
